# Effects of acupuncture and acupressure of the acupoint compared to the tendon on the blood circulation of human tendon in vivo

**DOI:** 10.1007/s00421-023-05277-2

**Published:** 2023-07-15

**Authors:** Keitaro Kubo, Ayaka Yasuda, Hiroyoshi Yajima, Miho Takayama, Nobuari Takakura

**Affiliations:** 1 Department of Life Science (Sports Sciences), The University of Tokyo, Komaba 3-8-1, Meguro-Ku, Tokyo, 153-8902 Japan; 2 Department of Acupuncture and Moxibustion, Tokyo Ariake University of Medical and Health Sciences, Ariake 2-9-1, Koto-Ku, Tokyo, 135-0063 Japan

**Keywords:** Blood volume, Oxygen saturation, Achilles tendon, Human

## Abstract

**Purpose:**

The purpose of this study was to compare the effects of acupuncture and acupressure of acupoints on tendon blood circulation with those of both types of stimulation of tendon itself.

**Methods:**

Before, during (except for acupressure), and after acupuncture and acupressure of the tendon and acupoint, blood circulation of the Achilles tendon was measured using red laser lights.

**Results:**

The blood volume of the treated and non-treated tendons increased after acupuncture of the tendon (effect of time p = 0.030), whereas that tended to increase after acupuncture of the acupoint (effect of time p = 0.063). In addition, no significant difference in the increases in blood volume was found among the four conditions, i.e., after acupuncture stimulation of the tendon and acupoint for the treated and non-treated tendons (p = 0.492). The blood volume of the treated tendon significantly increased after acupressure of the tendon (effect of time p < 0.001), but not of the acupoint (effect of time p = 0.260), whereas that of the non-treated tendon did not change after acupressure of both the tendon and acupoint.

**Conclusion:**

These results suggested that acupuncture of the tendon and acupoint acted centrally to enhance blood circulation of both the treated and non-treated tendons during the recovery period, whereas acupressure of the tendon locally increased blood circulation of the treated tendon only, but not the non-treated tendon and both the treated and non-treated tendons after acupressure of acupoint.

## Introduction

It is known that tendon disorders are linked to disturbances in tendon vasculature, which result in insufficiency in blood circulation (Jarvinen et al. 1997). As a result of local tissue hypoxia and inadequate nutrition brought on by decreased blood circulation within the tendons, tendon degeneration is likely to occur (Ahmed et al. 1998; Carr and Norris 1989; Stein et al. 2000; Zantop et al. 2003). In the medical field, acupuncture and acupressure are known to be effective in treating tendon disorders (e.g., Alim et al. 2018; Kleinhenz et al. 1999). The healing mechanisms of tendon disorders with these therapies included improved blood circulation of the tendons (Chen et al. 2001; Greve et al. 2012; Praxitelous et al. 2018). In fact, our studies showed that blood volume and oxygen saturation of the human tendons increased after acupuncture and acupressure (Kubo et al. 2010, 2011, 2020a, b; Yasuda et al. 2022). In all of these studies, acupuncture and acupressure were directly applied to the tendons. In the medical field, however, these therapies are generally performed at a distance from the affected area, i.e., the remote acupoint (Haker and Lunderberg 1990; Fink et al. 2002).

Many previous studies have shown that muscle and skin blood flow increased with acupuncture and acupressure of acupoints (Hsiu et al. 2010; Li et al. 2007; Litscher et al. 2002; Sandberg et al. 2003). To our knowledge, no studies animal or human studies have examined the effects of acupuncture and acupressure of remote acupoints on tendon blood circulation. Although the acupoints have not been fully elucidated, histochemical studies reported that acupoints were sites of dense concentrations of Aδ and C afferent sensory nerve fibers (Li et al. 2004). To apply our findings in a medical setting, we need to investigate the effects of acupuncture and acupressure of the acupoints, i.e., at points distant from the tendons, on the blood circulation of the human tendons in vivo.

From the results of animal experiments (Kagitani et al. 2005, 2010), acupuncture of acupoints on the lower leg has been shown to cause activation of Aδ and C fibers in the dorsal root ganglion of the lumbar region. In human experiments, acupuncture and acupressure of acupoints have been shown to decrease heart rate and alter the autonomic nervous system (as assessed by heart rate variability) (Arai et al. 2011; Haker et al. 2000; Matsubara et al. 2011; Nishijo et al. 1997). Our previous studies demonstrated that blood circulation of the tendons in the treated and non-treated sides increased after acupuncture and acupressure of the Achilles tendon (Kubo et al. 2011, 2020a, b). These results suggested that acupuncture and acupressure of tendons elicited a systemic response. In light of the above, acupuncture and acupressure of acupoints is also expected to produce a systemic reaction (i.e., an increase in tendon blood circulation in the treated and non-treated sides) through the sensory nerves distribution to the acupoints.

The purpose of this study was to compare the effects of acupuncture and acupressure of acupoints on tendon blood circulation with those of both types of stimulation of tendons. We hypothesized that acupuncture and acupressure of acupoint would enhance blood circulation of the tendon on both the treated and non-treated sides due to a systemic response during the recovery period after stimulation, as is the case with stimulation of the tendon, because of the dense concentration of sensory nerves at the acupoint as described above.

## Methods

### Participants

**Fig. 1:**
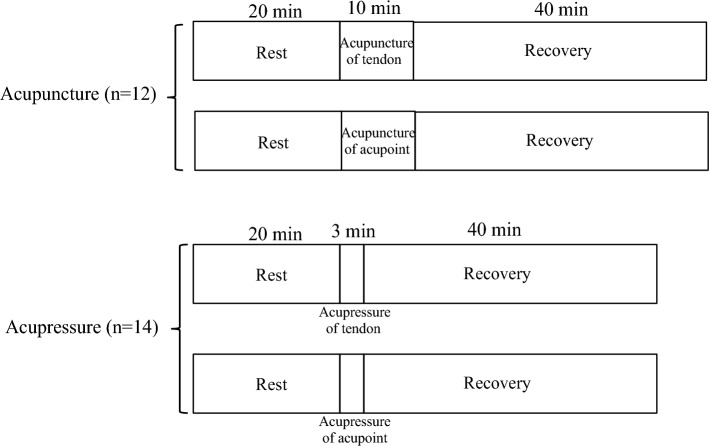
Experimental protocol (see Methods for a detailed description of the protocol)

The sample size was estimated according to our previous studies in which the increases in the blood volume of the Achilles tendon by acupuncture and acupressure were determined (0.63 ± 0.56 µmol·L^−1^ in Kubo et al. 2010, 0.65 ± 0.56 µmol·L^−1^ in Kubo et al. 2011, 0.87 ± 1.05 µmol·L^−1^ in Kubo et al. 2020a). Based on an α level of 0.05 and a power (1 – ß) of 0.8, it was shown that at least eleven participants per group were necessary for the present study. Twenty-six healthy volunteers (23 males and 3 females; age: 27.8 ± 9.7 years, height: 170.9 ± 6.7 cm, body mass: 69.2 ± 14.0 kg, mean ± SD) participated in this study. Twelve and fourteen volunteers participated in the acupuncture and acupressure experiments (Fig. 1). Exclusion criteria included a history of injuries and surgery on the Achilles tendon and cardiovascular diseases related to blood circulation. The participants were fully informed of the procedures to be utilized and the purpose of this study. Written informed consent was obtained from all participants. This study was approved by the office of the Department of Sports Sciences, The University of Tokyo, and complied with their requirements for human experimentation, and was performed by the Declaration of Helsinki. One part of the data in our previous study (n = 13, tendon blood circulation during and after acupuncture of the tendon in Kubo et al. 2020a) diverted to the data in the present study (n = 12; tendon blood circulation during and after acupuncture of the tendon), because 12 of the 13 participants in our previous study (Kubo et al. 2020a) took part in the measurement of the changes in tendon blood circulation with acupuncture of the acupoint due to scheduling limitations.

### Acupuncture and acupressure

The participants lay prone on a test bench to acclimate to the laboratory conditions for 15 min before the experiment. Initially, the participants lay in a prone position for a 20 min rest period (baseline). After that, a needle was inserted (acupuncture stimulation experiment) or acupressure was applied (acupressure stimulation experiment) to the right Achilles tendon or acupoint (BL57 Chengshan). BL57 was chosen as an acupoint and was located in the myotendinous junction of the triceps surae, which is continuous with the Achilles tendon (Zhang et al. 2004). After the needle was removed or the acupressure was stopped, the participants remained relaxed in the same position for 40 min. Each participant received two treatments (stimulation of tendon and acupoint) on two separate days, with at least one week between sessions and no longer than two weeks separating the two sessions. The entire experimental protocol is described in Fig. 1. The order of the two experimental conditions was randomized for each participant.

**Fig. 2:**
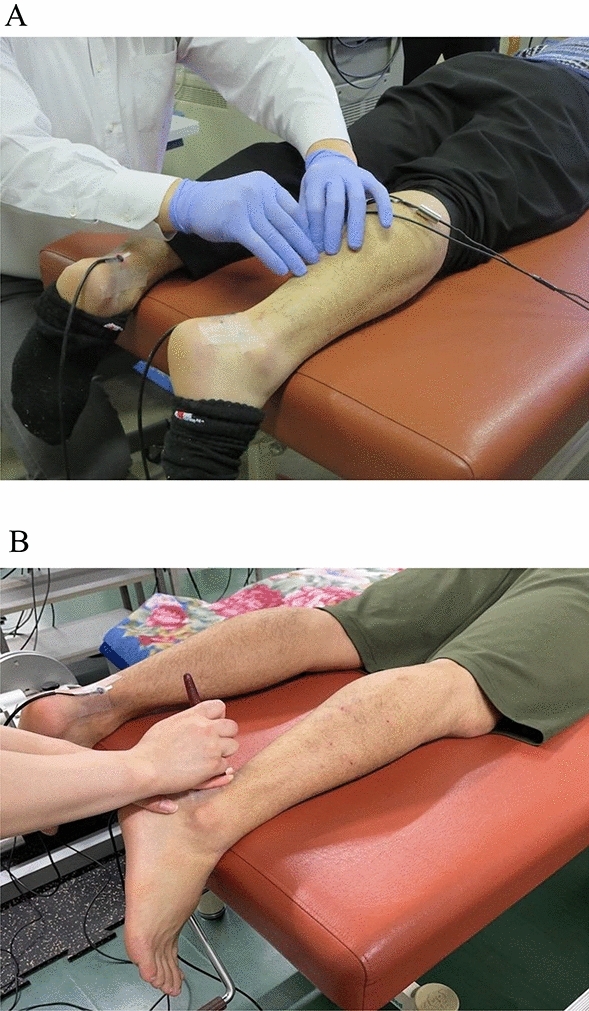
Views of acupuncture (**A**) and acupressure (**B**) stimulation experiments

*Acupuncture* Acupuncture was performed by one of the authors (H.Y.), an experienced (more than 25 years) licensed acupuncturist (Fig. 2A). A stainless steel needle of 0.16 mm in diameter and 40 mm in length was inserted vertically into the skin at 40 mm proximal to the calcaneus (stimulation of the tendon experiment) or the acupoint (stimulation of the acupoint experiment). To locate the acupoint (BL57), ultrasonography (SSD-3500, Aloka, Tokyo, Japan) was used to identify the boundary between the medial and lateral gastrocnemius muscles and the myotendinous junction between the Achilles tendon and gastrocnemius muscle. After insertion of the needle to a targeted depth (4 mm for the tendon, 10 mm for the acupoint), it was left for 5 min without manipulation (Acu-1). Then, the tip of the needle was moved up and down from the targeted depth (up-and-down manipulation) at an approximately 1-mm amplitude and 2 Hz for 3 min (Acu-2). During Acu-2, the needle tip was moved at an approximately constant speed and frequency with the aid of a metronome, and the insertion depth was confirmed visually. After this technique, the needle was left for 2 min without manipulation (Acu-3).

*Acupressure* Acupressure was applied by one of the authors (A.Y.) (Fig. 2B). In the present study, we used a modified wooden stick (Tubo, Lieb Corporation, Japan) with a force sensor to monitor the pressing force during acupressure (Yasuda et al. 2022). This stick was pressed perpendicularly to the skin at 40 mm proximal to the calcaneus (stimulation of the tendon experiment) or the acupoint (stimulation of the acupoint experiment). Acupressure was performed at a frequency of 1.5 Hz for 3 min with a pressing force of 50 N, as described in our previous study (Yasuda et al. 2022).

### Blood circulation of the Achilles tendon

During the experimental period, the blood circulation (oxyhemoglobin; Oxy, deoxyhemoglobin; Deoxy, total hemoglobin; THb, oxygen saturation; StO_2_) of the Achilles tendon was measured on both the treated and non-treated sides using a previously described procedure (Kubo et al. 2011, 2020a, b). First, a probe (SF-DS, Omega Wave, Tokyo, Japan) was positioned at 20 mm proximal to the calcaneus to measure the blood circulation of the tendon using red laser lights (BOM-L1TRSF, Omega Wave, Tokyo, Japan). This instrument uses three red laser lights (635, 650, and 690 nm) and calculates the relative tissue levels of Oxy, Deoxy, and THb (corresponding to the blood volume) according to the Beer-Lambert law. Oxy, Deoxy, THb, and StO_2_ at a specific depth (measurement depth of 3–5 mm) of the tissue could be measured by changing the location of the two detectors (Kashima 2003; Kubo et al. 2008).

In the present study, the units of Oxy, Deoxy, and THb are expressed as µmol / l, although this does not represent the actual physical volume. StO_2_ was calculated using the formula from Oxy and THb values: StO_2_ (%) = 100 · Oxy · THb^−1^. Our data were input into a personal computer at a sampling frequency of 10 Hz via an A/D transducer (Power Lab, AD Instruments, Australia). The mean values over a given duration (Acu-1, Acu-2, Acu-3, and every 10 min during the recovery period) were calculated using analytical software (Chart ver. 7.1, AD Instruments, Australia). Oxy, Deoxy, THb, and StO_2_ data are presented as the increase from the resting level. The repeatability of measurement of tendon blood circulation (Oxy, Deoxy, THb, and StO_2_) had been investigated in our previous studies (Kubo et al. 2008).

### Statistical analysis

Values are reported as means ± SD. Two-way (site x time) analysis of variance (ANOVA) with repeated measures was used to detect significant differences in the measured variables from the resting level. One-way ANOVA was used to detect a significant difference in mean changes in THb during the recovery period among the four conditions (the treated and non-treated tendons due to the treatments of the tendon and acupoint). The F ratios for main effects and interactions were considered significant at *p* < 0.05. Significant differences among means at *p* < 0.05 were detected using the Tukey’s HSD *post-hoc* test. In ANOVA, Mauchly's sphericity test was performed to assess the homogeneity of variance. Greenhouse–Geisser correction was applied where the assumption of sphericity was violated. The effect size was calculated using partial eta-squared (*pη*^2^) for two-way ANOVA. Pearson product-moment correlations were computed to assess the associations among the measured variables. The level of significance was set at *p* < 0.05.

## Results

**Fig. 3:**
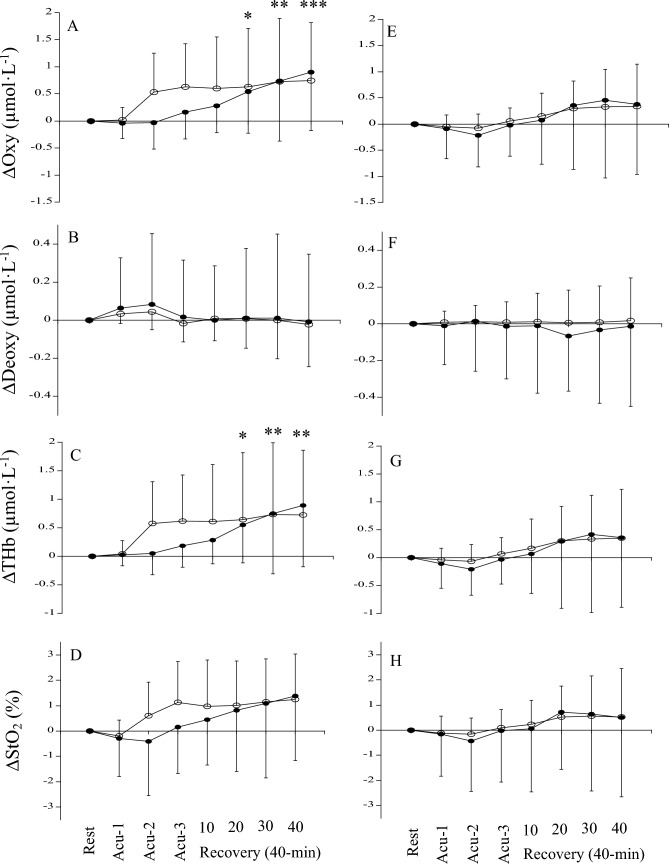
**A**–**D** The changes in oxyhemoglobin (Oxy), deoxyhemoglobin (Deoxy), total hemoglobin (THb), and oxygen saturation (StO_2_) of treated (open circle) and non-treated (closed circle) tendons during acupuncture of the tendon and recovery periods. **E**–**H** The changes in oxyhemoglobin (Oxy), deoxyhemoglobin (Deoxy), total hemoglobin (THb), and oxygen saturation (StO_2_) of treated (open circle) and non-treated (closed circle) tendons during acupuncture of the acupoint and recovery periods. *Significantly different from the resting level (*p < 0.05, **p < 0.01, ***p < 0.001)

The changes in the blood circulation of the treated and non-treated tendons due to acupuncture of the tendon are shown in Fig. 3A–D. For Oxy, THb, and StO_2_, the effect of time was significant (*p* = 0.024 *pη*^2^ = 0.299 for Oxy, *p* = 0.030 *pη*^2^ = 0.294 for THb, *p* = 0.024 *pη*^2^ = 0.264 for StO_2_), although the effects of site (*p* = 0.377 *pη*^2^ = 0.071 for Oxy, *p* = 0.514 *pη*^2^ = 0.040 for THb, *p* = 0.433 pη^2^ = 0.057 for StO_2_) and the interaction between site and time (*p* = 0.081 *pη*^2^ = 0.211 for Oxy, *p* = 0.144 *pη*^2^ = 0.169 for THb, *p* = 0.160 *pη*^2^ = 0.148 for StO_2_) were not significant. The *post-hoc* analysis identified significant increases for Oxy and THb at 20, 30, and 40 min points during the recovery period, although that did not identify significant differences for StO_2_ at any point. For Deoxy, the effects of site (*p* = 0.853 *pη*^2^ = 0.003) and time (*p* = 0.377 *pη*^2^ = 0.084) and the interaction between site and time (*p* = 0.851 *pη*^2^ = 0.011) were not significant.

The changes in the blood circulation of the treated and non-treated tendons due to acupuncture of the acupoint are shown in Fig. 3E–H. For Oxy, Deoxy, THb, and StO_2_, the effects of site (*p* = 0.949 *pη*^2^ = 0.000 for Oxy, *p* = 0.739 *pη*^2^ = 0.010 for Deoxy, *p* = 0.848 *pη*^2^ = 0.004 for THb, *p* = 0.931 *pη*^2^ = 0.001 for StO_2_) and the interaction between site and time (*p* = 0.757 *pη*^2^ = 0.021 for Oxy, *p* = 0.702 *pη*^2^ = 0.027 for Deoxy, *p* = 0.791 *pη*^2^ = 0.015 for THb, *p* = 0.811 *pη*^2^ = 0.017 for StO_2_) were not significant. The effect of time for Oxy (*p* = 0.052 *pη*^2^ = 0.253), THb (*p* = 0.063 *pη*^2^ = 0.242), and StO_2_ (*p* = 0.109 *pη*^2^ = 0.195) tended to be significant, although that for Deoxy was not (*p* = 0.691 *pη*^2^ = 0.019).

**Fig. 4:**
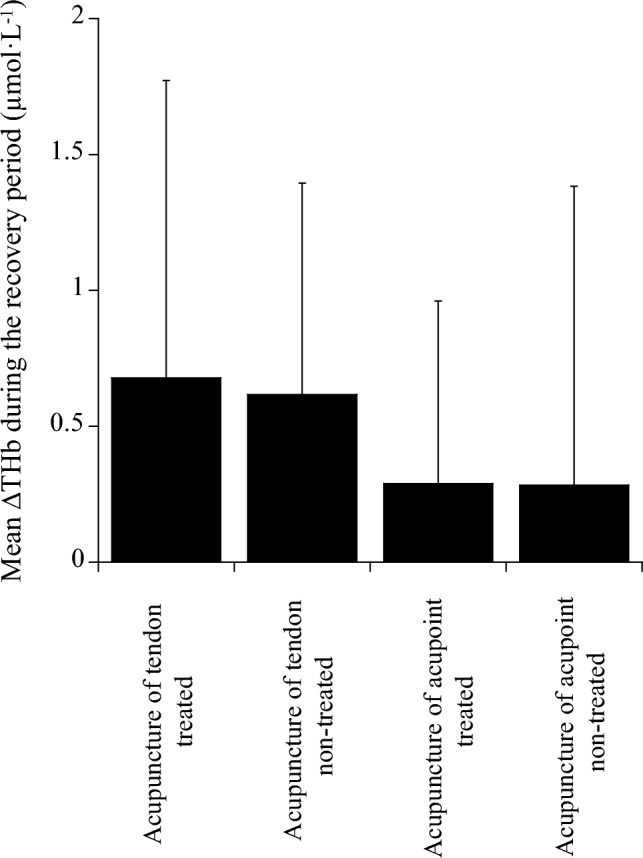
Comparison of mean ∆THb during the recovery period after acupuncture among the four conditions

**Fig. 5:**
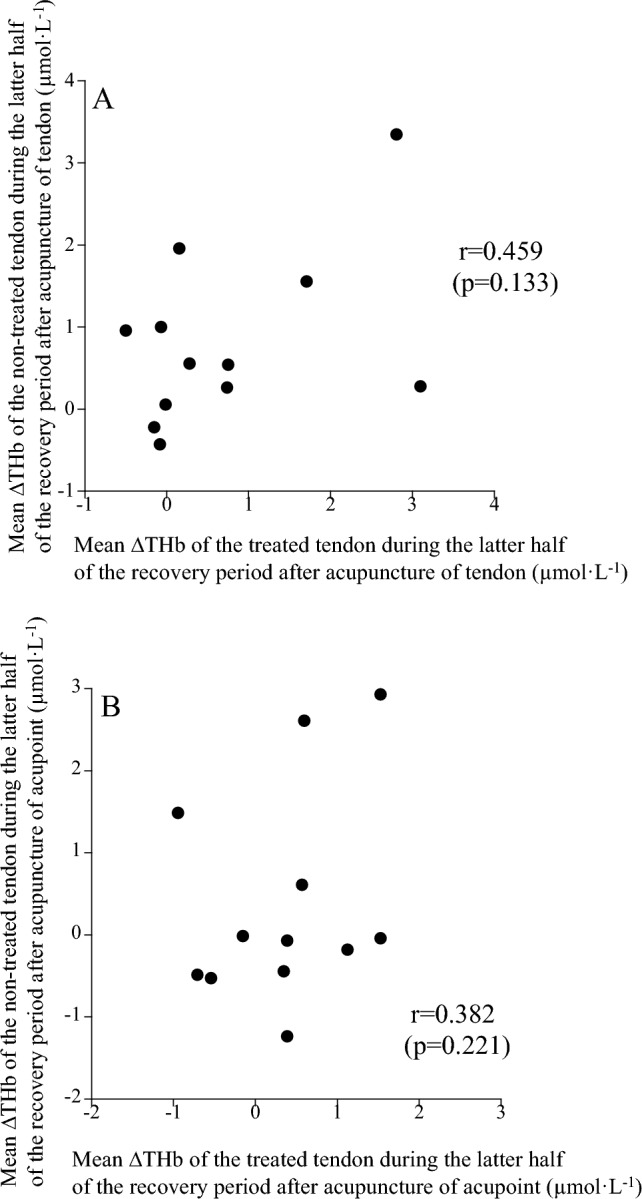
Relationships between mean ∆THb of treated and non-treated tendons during the latter half of the recovery period after acupuncture of the tendon (**A**) and acupoint (**B**)

No significant difference in the changes in THb during the recovery period after acupuncture of the tendon and acupoint for the treated and non-treated tendons was found among the four conditions (*p* = 0.492 *pη*^2^ = 0.069; Fig. 4). The changes in THb on the treated tendons during the latter half of the recovery period (21–40 min) were not correlated with the changes in THb on the non-treated tendons after acupuncture of the tendon (*r* = 0.459, *p* = 0.133) and acupoint (*r* = 0.382, *p* = 0.221) (Fig. 5).

**Fig. 6:**
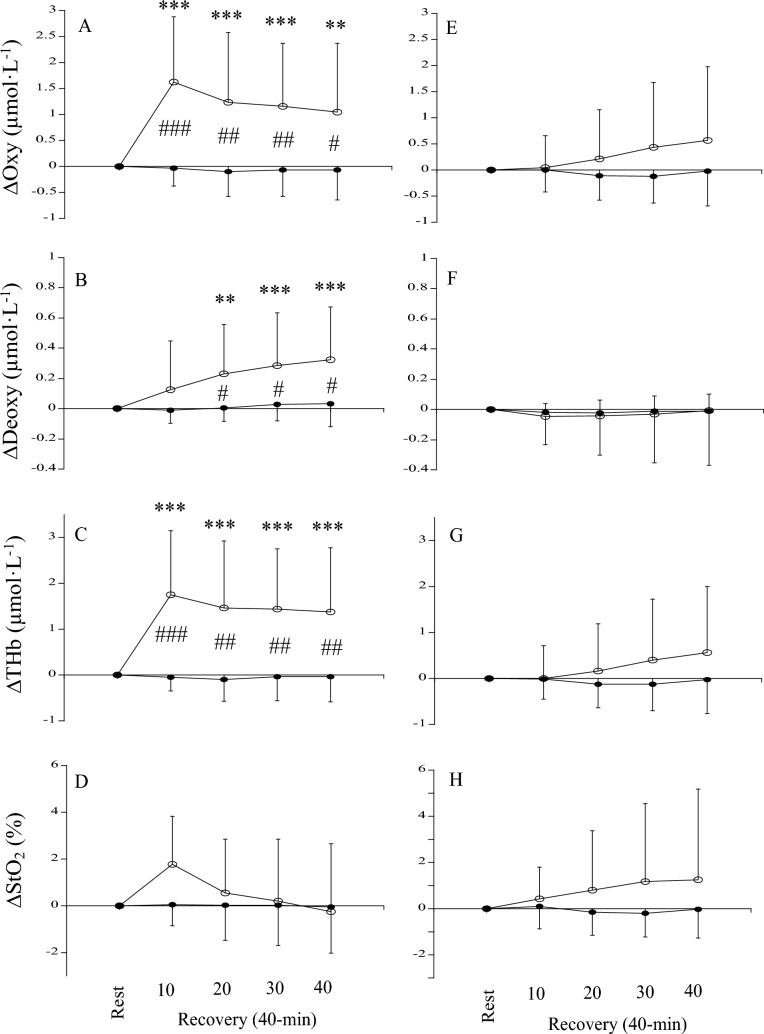
**A**–**D** The changes in oxyhemoglobin (Oxy), deoxyhemoglobin (Deoxy), total hemoglobin (THb), and oxygen saturation (StO_2_) of treated (open circle) and non-treated (closed circle) tendons before (rest) and after acupressure of the tendon (recovery). **E**–**H** The changes in oxyhemoglobin (Oxy), deoxyhemoglobin (Deoxy), total hemoglobin (THb), and oxygen saturation (StO_2_) of treated (open circle) and non-treated (closed circle) tendons before (rest) and after acupressure of the acupoint (recovery). *Significantly different from the resting level (**p < 0.01, ***p < 0.001) ^#^significantly different from the non-treated side (^#^p < 0.05, ^##^p < 0.01, ^###^p < 0.001)

The changes in the blood circulation of the treated and non-treated tendons due to acupressure of the tendon are shown in Fig. 6A–D. For Oxy, Deoxy, and THb, the effects of site (*p* = 0.002 *pη*^2^ = 0.538 for Oxy, *p* = 0.030 *pη*^2^ = 0.313 for Deoxy, *p* = 0.001 *pη*^2^ = 0.574 for THb) and time (*p* = 0.002 *pη*^2^ = 0.384 for Oxy, *p* = 0.002 *pη*^2^ = 0.389 for Deoxy, *p* < 0.001 *pη*^2^ = 0.437 for THb) and the interaction between site and time (*p* < 0.001 *pη*^2^ = 0.454 for Oxy, *p* = 0.014 *pη*^2^ = 0.318 for Deoxy, *p* < 0.001 *pη*^2^ = 0.490 for THb) were significant. Regarding the treated tendon due to acupressure of the tendon, the *post-hoc* analysis identified significant increases for Oxy, Deoxy, and THb at all time points during the recovery period (except for Deoxy at 10 min). For StO_2_, the effect of site (*p* = 0.525 *pη*^2^ = 0.032) and the interaction between site and time (*p* = 0.120 *pη*^2^ = 0.161) were not significant. Although the effect of time for StO_2_ was significant (*p* = 0.031 *pη*^2^ = 0.229), the *post-hoc* analysis did not identify significant differences at any point.

The changes in the blood circulation of the treated and non-treated tendons due to acupressure of the acupoint are shown in Fig. 6E–H. For Oxy, Deoxy, THb, and StO_2_, the effects of site (*p* = 0.233 *pη*^2^ = 0.107 for Oxy, *p* = 0.799 *pη*^2^ = 0.005 for Deoxy, *p* = 0.280 *pη*^2^ = 0.089 for THb, *p* = 0.204 *pη*^2^ = 0.121 for StO_2_) and time (*p* = 0.273 *pη*^2^ = 0.095 for Oxy, *p* = 0.620 *pη*^2^ = 0.028 for Deoxy, *p* = 0.260 *pη*^2^ = 0.099 for THb, *p* = 0.458 *pη*^2^ = 0.054 for StO_2_) and the interaction between site and time (*p* = 0.154 *pη*^2^ = 0.140 for Oxy, *p* = 0.854 *pη*^2^ = 0.007 for Deoxy, *p* = 0.175 *pη*^2^ = 0.130 for THb, *p* = 0.235 *pη*^2^ = 0.107 for StO_2_) were not significant.

**Fig. 7:**
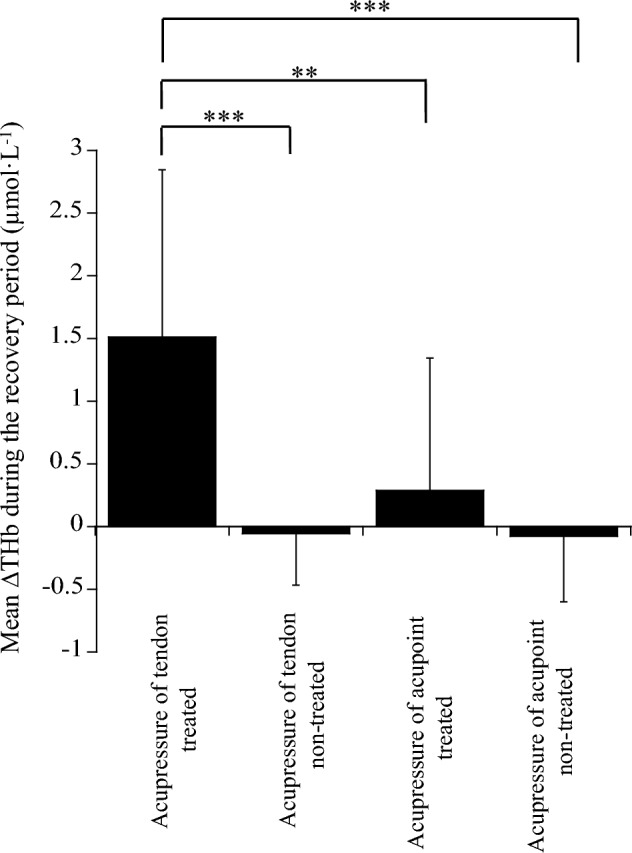
Comparison of mean ∆THb during the recovery period after acupressure among the four conditions. *Significantly different among the conditions (**p < 0.01, ***p < 0.001)

The change in THb during the recovery period after acupressure of the tendon for the treated tendon was significantly greater than that of the tendon (*p* < 0.001) and acupoint (*p* < 0.001) for the non-treated tendon and that of the acupoint for the treated tendon (*p* = 0.005) (Fig. 7).

## Discussion

Consistent with our previous studies (Kubo et al. 2010, 2011, 2020a, b), acupuncture stimulation of the tendon showed a rapid change in blood circulation on the treated tendon during acupuncture with up-and-down manipulation (Acu-2), with no change on the contralateral side (non-treated tendon). These changes during acupuncture with up-and-down manipulation are thought to be due to the release of neurotransmitters (e.g., substance P), resulting in axon reflex (Kashiba and Ueda 1991; Sato et al. 2000). During the recovery period after removal of the needle, tendon blood volume significantly increased on both the treated and non-treated sides (Fig. 3C). Unfortunately, no significant correlation was found between tendon blood volume on the treated and non-treated sides (Fig. 5), although several of our previous studies found significant correlations between the two (Kubo et al. 2011, 2020b). However, regarding acupuncture of the tendon, the increase in tendon blood volume of the treated tendon was significantly correlated with that of the non-treated one, if the outlier of one participant was excluded (*r* = 0.729, *p* = 0.011; Fig. 5A). Reasons for the discrepancies may include the number of participants (n = 17 in Kubo et al. 2011, n = 21 in Kubo et al. 2020b, n = 12 in the present study). In any case, the present results may be attributed to central effects via cholinergic vasodilator fibers (Inoue et al. 2001). On the other hand, both during and after acupuncture stimulation, Oxy increased, and Deoxy did not change (Fig. 3). These results are presumably due to increased blood inflow to the tendon via the axon reflex (primarily during acupuncture with up-and-down manipulation) and cholinergic vasodilator fibers (primarily during the recovery period). Thus, acupuncture of tendons altered blood circulation of the treated tendon by peripheral and central effects and the non-treated tendon by central effect.

Acupuncture of the acupoint did not change tendon blood circulation during acupuncture with up-and-down manipulation, unlike acupuncture of the tendon. Our inference that the increased blood circulation in the Achilles tendon with a needle insertion to this tendon is due to the axon reflex (Kashiba and Ueda 1991; Sato et al. 2000) is supported by the present finding that blood circulation in the Achilles tendon did not increase with a needle insertion to the acupoint. On the other hand, during the recovery period after the removal of the needle, blood volume tended to increase on both treated and non-treated tendons (effect of time *p* = 0.063; Fig. 3G). This result suggested that acupuncture of the acupoint acted to enhance blood inflow to tendons by cholinergic vasodilator fibers through the central nervous system (Inoue et al. 2001). However, the increase in tendon blood volume due to acupuncture of the acupoint did not show a significant correlation between the treated and non-treated sides as in the case of acupuncture of the tendon in the latter half of the recovery period (Fig. 5). Therefore, there may be a greater abundance of sensory nerves (related to cholinergic vasodilation) in the Achilles tendon than in the acupoint (Chengshan, BL57). Regardless, further studies using isolated animal tendons are needed to clarify this point.

We recently reported that acupressure of the tendon increased blood volume on both the treated and the non-treated tendons (Kubo et al. 2020a). In the present study, however, no change in blood volume on the non-treated tendon was observed. In this previous study (Kubo et al 2020a), no significant correlation was found between the increase in blood volume on the treated and non-treated tendons as in the case of acupuncture of tendons (data not shown in Kubo et al. 2020a). Furthermore, in Yasuda et al. (2022), we obtained a similar result in which blood volume on the non-treated tendon did not change when acupressure was applied to the Achilles tendon (data not shown in Yasuda et al. 2022). Although the reasons for these discrepancies in results are unknown, the possible reason is the method of acupressure, i.e., 5-mm amplitude and 1.5 Hz in Kubo et al. (2020a) and 50 N and 1.5 Hz in the present study. In any case, it can be said that, unlike acupuncture, acupressure in this study does not produce centrally mediated changes in tendon blood circulation. Regarding the difference in hemodynamic changes caused by both types of stimulation, it is speculated that the nociceptive stimulation of tissues by acupuncture causes peripheral vasodilatation through the autonomic nervous system at the spinal level by ascending Aδ and C fibers (Chae et al. 2013). Furthermore, unlike acupuncture, acupressure of the tendon increased Deoxy on the treated side, resulting in no significant increase in StO_2_. Therefore, it is likely that acupressure of the tendon alters tendon blood circulation by enhancing the metabolism of the treated tendon, which is due to a local rather than a systemic response.

Acupressure of the acupoint did not produce changes in blood circulation on either the treated or non-treated tendons, although acupuncture of the acupoint tended to change tendon blood circulation. These results indicated that the sensory nerves present in acupoints could be stimulated by acupuncture but not by acupressure. In this regard, it has been pointed out that the excitation by nociceptive stimulation with acupuncture may ascend Aδ and C fibers and cause a vasodilating effect via the autonomic nervous system at the level of the spinal cord (Pomeranz and Chui 1976; Pomeranz and Warma 1988). Thus, the microscopic tissue damage caused by acupuncture may be related to differences between acupuncture and acupressure in their effects on tendon blood circulation.

Based on the results of this study, in acupuncture, when there is pain in the affected area immediately after the injury, therapeutic effects can be expected by applying the treatment to acupoints that are distant from the affected area. Acupuncture treatment on Chengshan (BL57), the acupoint employed in the present study, has actually been shown to be effective in the treatment of Achilles tendon disorders (Zhang et al. 2017). In addition, experiments with animals reported that acupuncture with a low voltage electrical current at BL57 increased the concentration of collagen and the collagen fibril diameters in rat Achilles tendons, indicating the strengthening of the tendons (Almeida et al. 2012, 2015). Unfortunately, it is inferred that acupressure of acupoints is not effective in curing tendon disorders. However, acupressure may be useful as maintenance to prevent injury by increasing the metabolism of the tendon.

In the present study, we must draw attention to the limitations associated with the methodology followed. Firstly, the locations of acupoints are defined (by WHO) by the bone, muscle, blood vessels, etc. as indicators, but the locations as acupoints in individuals may differ from the difference of these anatomical landmarks. If so, differences in the location of acupoints in different individuals may affect the results of acupuncture and acupressure of acupoints. In this study, we used ultrasonography to define the stimulation location while confirming the boundary between the medial gastrocnemius muscle and lateral gastrocnemius muscle and the muscle–tendon junction of the gastrocnemius muscle with images. Secondly, changes in the blood circulation of the Achilles tendon were only measured 40 min after both acupuncture and acupressure. Further observation over a longer post-stimulation period is needed to examine the therapeutic effect on tendon disorders. Thirdly, all of the participants in this study were healthy and were not patients with tendon disorders. It cannot be ruled out that changes in tendon blood circulation in response to acupuncture or acupressure may differ between healthy participants and patients with tendon disorders. Future studies should be conducted on patients with tendon disorders to verify the therapeutic effects of acupuncture and acupressure treatment.

In conclusion, the main results of the present study were that (1) acupuncture of tendon and acupoint acted centrally to enhance blood circulation of both the treated and non-treated tendons during the recovery period after stimulation, and (2) acupressure of tendon locally increased tendon blood circulation by enhancing the metabolism of the treated tendon only, whereas acupressure of acupoint did not alter tendon blood circulation.

## Abbreviations

Acu: Acupuncture
ANOVA: Analysis of variance
Deoxy: Deoxyhemoglobin
Oxy: Oxyhemoglobin
pη^2^: Partial eta-squared
SD: Standard deviation
StO_2_: Oxygen saturation
THb: Total hemoglobin
